# Neurocognition and mean radiotherapy dose to vulnerable brain structures: new organs at risk?

**DOI:** 10.1186/s13014-023-02324-2

**Published:** 2023-08-11

**Authors:** Helena Söderström, Angelica Walfridsson, Ulla Martinsson, Ulf Isacsson, Karin Brocki, Johan Lundin Kleberg, Gustaf Ljungman

**Affiliations:** 1 Present Address: Department of Women’s and Children’s Health, Uppsala University, Uppsala, Sweden; 2 Department of Hematology and Oncology, Uppsala University Hospital, Uppsala, Sweden; 3 Department of Immunology, Genetics and Pathology, Uppsala University, Uppsala, Sweden; 4 Department of Psychology, Uppsala University, Uppsala, Sweden; 5 Department of Psychology, Stockholm University, Stockholm, Sweden; 6 Department of Clinical Neuroscience, Karolinska Institutet, Stockholm, Sweden

**Keywords:** Pediatric brain tumor, Neurocognition, Radiotherapy doses, Organs at risk

## Abstract

**Background:**

Children with brain tumors are at high risk of neurocognitive decline after radiotherapy (RT). However, there is a lack of studies on how RT doses to organs at risk (OARs) impacts neurocognition. The aim of this study was to examine dose-risk relationships for mean RT dose to different brain structures important for neurocognitive networks. We explored previously established OARs and potentially new OARs.

**Methods:**

A sample of 44 pediatric brain tumor survivors who had received proton and/or photon RT were included. Correlations between mean RT doses to OARs and IQ were analyzed. Previously established OARs were cochleae, optic chiasm, optic nerve, pituitary gland, hypothalamus, hippocampus and pons. Potential new OARs for RT-induced neurocognitive decline were cerebellum, vermis and thalamus.

**Results:**

Mean RT dose to different OARs correlated with several IQ subtests. Higher mean RT dose to cochleae, optic nerve, cerebellum, vermis and pons was correlated with lower performance on particularly full-scale IQ (FIQ), Perceptual Reasoning (PRI), Working Memory (WMI) and Processing Speed Index (PSI). Higher mean RT dose to hippocampus correlated with lower performance on processing speed and working memory. For those receiving whole brain RT (WBRT), higher mean RT dose to the pituitary gland correlated with lower performance on working memory.

**Conclusion:**

A high dose-risk correlation was found between IQ subtests and mean RT dose in established and potential new OARs. Thus, in the lack of validated dose constraints for vulnerable brain structures, a parsimonious approach in RT planning should be considered to preserve neurocognitive networks.

**Supplementary Information:**

The online version contains supplementary material available at 10.1186/s13014-023-02324-2.

## Background

Younger age at RT, higher dose to normal brain tissue, RT dose and volume of the tissue being exposed are associated with higher risk for neurocognitive decline [1, 2]. Children are especially vulnerable to RT-induced neurocognitive decline as RT can impair development of white and grey matter, cause neuroinflammation, and inhibit neurogenesis and synaptic plasticity [3–6]. There are several confounding risk factors that can impact neurocognition such as increased intracranial pressure, tumour size, surgery, and chemotherapy that has been investigated in our previous study [7]. Prescribed doses or planning target volumes are common measures for investigating neurocognitive late effects [7, 8]. In our previous study, we found that higher dose to planning target volume was moderately correlated with one IQ subtest that measures working memory [7]. However, physical mean dose metrics account for dose heterogeneity and may be a better predictor for RT-induced neurocognitive decline [8, 9].

Radiation dose to OARs can impact neural structures that play an important role in neurocognition and disrupt functional brain networks during development. For instance, radiation dose to cochleae can cause hearing loss [10–12], which in turn has been associated with intellectual impairment and lower academic performance in children treated for medulloblastoma [13]. Optic chiasm and the optic nerve are also defined as OARs due to the risk of RT-induced optic neuropathy and can impact neurocognition through connections to the central nervous system (CNS) [12, 14, 15].

Cerebellum, vermis and thalamus are not typically defined as OARs although many neurocognitive networks are connected with these brain structures. The cerebellum has a complex interaction with the cerebral cortex through the cerebro-cerebellar loops (the cortico-ponto-cerebellar pathway and cerebello-thalamo-cortical pathway) [16, 17]. The cerebellum plays an important role in sensorimotor function and neurocognition such as working memory, language, and executive function [18]. A higher RT dose to the cerebellum in ependymoma patients is associated with a decline in multiple neurocognitive domains such as IQ and academic achievement (reading, math, and spelling) [19, 20]. The vermis is sensitive to higher radiation dose since it has several connectional networks to the brain such as pons, hippocampus and limbic structures. Lesions in the vermis are associated with neurocognitive decline and social-emotional behavioral problems [16, 18]. The cerebellum has connections with pons and thalamus [16, 17]. The thalamus is a widespread broader cortico-subcortical network, and injuries can explain late effects with visual attention and memory [21]. Hypothalamic-pituitary (HP) disorders are common after high-dose RT. Lower performance on IQ and memory has been associated with HP disorders [22].

The developing hippocampus is nowadays established as an OAR and is highly sensitive to cranial RT in pediatric brain tumor patients. Lowering the RT doses [12, 23] and also avoidance of the hippocampi during WBRT are recommended as this has been associated with the preservation of memory and higher quality of life [24]. Further, there are few brain metastases in the hippocampus which indicates that a sparing approach is possible [25].

With proton RT greater dose sparing can often be achieved in OARs, compared to photon RT [6, 26, 27]. Proton RT is associated with overall better neurocognitive performance and IQ scores compared to photon RT [1, 2, 28]. Improved sparing of radiation dose to different OARs can be achieved with intensity-modulated proton therapy (IMPT). A sparing approach to OARs can also be achieved with photon RT, e.g. through intensity-modulated radiation therapy (IMRT) or volumetric-modulated arc therapy (VMAT) [1, 26, 29]. Beneficial sparing with IMPT compared to IMRT has been demonstrated for different ages in medulloblastoma and ependymoma patients with improved sparing of critical structures such as cochlea, optic nerve, brainstem and pituitary gland [26].

In summary, few studies have investigated a dose-risk relationship between mean RT dose to different OARs and neurocognition. The overall aim of this study was to examine dose-risk relationships between mean RT doses to different brain structures important for neurocognitive networks. We hypothesized that mean RT dose to previously established OARs and to some potentially new OARs (cerebellum, vermis and thalamus) would be correlated with neurocognitive decline as measured by IQ measurements.

## Methods

### Study population

Inclusion criteria were: children treated with RT for brain tumor in childhood at a tertiary care Children’s University Hospital in Sweden (January 2003–June 2015), alive at the time of the data collection, with five years or more elapsed after diagnosis, and with access to RT treatment plans. A total of 44 children who had received photon and/or proton RT were included, 17 children received WBRT (where WBRT was generally part of craniospinal irradiation), 21 children received photon RT, 12 proton and photon RT, and 11 proton RT. The most common tumors were embryonal, ependymal, astrocytic and germ cell tumors. Mean age when they received RT was 10 years (range 3–17 years). For more details about the study population we refer to a previous publication [7] and Additional file 1: Table S1.

Radiation treatment plans, medical and neuropsychological data were collected from the medical records, and the Swedish Childhood Cancer Registry including the Radtox Quality Registry (a national RT registry for children).

### Organs at risk

Previously established OARs and potential new OARs for RT-induced neurocognitive decline were included. The delineated previously established OARs were: left and right cochleae, optic chiasm, left and right optic nerve, pituitary gland, left and right hypothalamus, left and right hippocampus and pons. Potential new OARs were: cerebellum, vermis and thalamus. These structures and pons were delineated as potential new OARs since many neurocognitive networks are connected to these brain structures [18].

### Radiotherapy

Photon RT was delivered by linear accelerators and with suitable beam qualities at the radiation department at Uppsala University Hospital in Sweden. Proton RT was delivered at the former The Svedberg laboratory, Uppsala University, applying a fixed horizontal beam with a maximal energy of 180 MeV. All OARs were outlined by the same author (AW) on computed tomography (CT) images of the brain. OARs were outlined according to the Radiation Therapy Oncology Group guidelines [30] and the European Society of Radiotherapy and Oncology guidelines [31]. Since CT images were performed over a period of time, the quality and scan parameters shifted. Most images were performed with 3 mm slice thickness, with a range of 2 mm to 16 mm. All investigations were performed using contrast injections. Very small structures, e. g. hypothalamus and cochleae, were delineated with a margin to the real anatomical volume.

Contouring and dose calculation was performed in two different treatment planning systems TMS (Treatment Management System version 6.1ASP1) and Oncentra (version 4.5.3) depending on which system the children had their treatment planned earlier. After delineating and calculating the doses to the OARs, the doses were extracted to the OARs for each plan and for each child. Physical dose metrics were calculated regarding the mean dose, which is the average dose to the defined structures. The number of treatment plans varied between the children from one to five, and mean dose for the whole treatment was calculated by summarizing the mean dose for each individual plan. For children receiving re-irradiation after their primary treatment, no time correction has been used when summarizing doses, since the time gap was considered negligible in comparison to the very long follow-up time.

All radiation doses were converted to the equivalent dose given in 2 Gray (Gy) fractions, with the linear-quadratic model, using an alfa/beta ratio of 3, to make it possible to compare doses and their biological effect. Proton RT doses were based on a proton specific relative biological effectiveness relative to high-energy photons (RBE 1.1).

### Neurocognitive variables

IQ measurements were collected from the clinical neuropsychological records and test protocols. During the current study period, neuropsychological assessment screening was used as a clinical standard [7, 32]. The latest assessment for each child was used in the analysis of neurocognitive function after RT doses to different OARs. Depending on age and different time points when the neuropsychological screening was assessed, different versions of the Wechsler scales were used: Wechsler Preschool and Primary Scale of Intelligence, third and fourth edition [33], Wechsler Intelligence Scale for Children third, fourth and fifth edition [34–36] and Wechsler Adult Intelligence Scale third and fourth edition [37, 38]. All IQ index FIQ, Verbal Reasoning Index (VRI), PRI, WMI and PSI were analyzed, with a normative mean of 100 and a standard deviation (SD) of 15. When all IQ subtests had not been administered, an estimated score was calculated for each IQ index (as described in the manuals). The most common administered subtests were further analyzed and scores were converted to age standardized scores (SS) with a normative mean of 10 and SD of 3. Included subtests were similarities, vocabulary, matrix reasoning, block design, digit span, letter-number-sequencing, coding, and symbol search.

### Statistical analyses

Spearman rank correlation coefficient (*r*_*s*_) was used to analyze correlations between different RT doses and neurocognitive function defined as IQ assessments. Correlations of *r*_*s*_ =  ± 0.7 to ± 1.0 were regarded as strong, *r*_*s*_ =  ± 0.4 to ± 0.6 as moderate and *r*_*s*_ =  ± 0.1 to ± 0.3 as weak [39]. One sample t-test compared the sample mean on IQ index to the normative mean scaled score of 100 (SD = 15) and IQ subtests to the normative mean scaled score of 10 (SD = 3). An alpha level of < 0.05 was regarded as significant. The statistical analyses were performed with the SPSS statistical program, version 28 [40].

## Results

The highest RT doses were given to cerebellum and pons. See Table 1, for means, standard deviations and range of radiation dose to OARs for the total cohort.

**Table 1:** Means, S.D.s, and range of radiotherapy dose to organs at risk for the total cohort (n = 44)

Organs at risk	M	S.D	Range
Cochlea right	21	20	0–55
Cochlea left	21	21	0–56
Optic nerve right	15	13	0–39
Optic nerve left	14	13	0–37
Hippocampus right	28	17	0–54
Hippocampus left	28	19	0–55
Pituitary gland	27	19	0–53
Cerebellum	28	23	0–65
Vermis	30	23	0–56
Pons	31	20	0–61
Chiasma	26	17	0–53
Thalamus	28	17	0–57
Hypothalamus right	29	19	0–54
Hypothalamus left	30	19	0–54

Neuropsychological assessment after RT was performed in 35 children (80%). Sample size varied for the different IQ subtests (n = 23–34; see Table 2 for descriptives). Mean performance for this clinical cohort was lower than the standardized mean on FIQ (t =  − 2.96, *P* = 0.006), VRI (t =  − 2.12, *P* = 0.04), WMI (t =  − 3.87, *P* < 0.001), and PSI (t =  − 5.16, *P* < 0.001). Mean performance for the study population was lower than the scaled score mean on vocabulary (t =  − 4.02, *P* < 0.001), digit span (t =  − 3.30, *P* = 0.002), digit span forward (t =  − 2.83, *P* = 0.008), letter-number-sequencing (t =  − 2.38, *P* = 0.03), coding (t =  − 5.93, *P* < 0.001), and symbol search (t =  − 4.21, *P* < 0.001).

**Table 2:** Means, medians, S.D.s, and range on IQ measurements after radiotherapy (9–139 months)

Cognitive measures	After radiotherapy (n = 35)				
	n	M	Mdn	S.D	Range
Full scale IQ**	29	90.52	89.00	17.28	61–123
VRI*	30	93.73	91.50	16.16	67–136
Similarities	33	9.73	9.00	3.45	4–18
Vocabulary***	34	8.15	8.50	2.69	2–14
PRI	30	97.73	97.50	18.84	67–129
Matrix reasoning	32	9.94	9.50	3.28	4–17
Block design	32	9.91	10.00	3.16	2–17
WMI***	30	88.40	89.00	16.42	56–132
Digit span**	34	8.44	9.00	2.78	3–15
Forward**	31	8.48	9.00	3.00	3–14
Backward	31	9.06	9.00	3.00	3–15
Letter-number-sequencing*	20	8.05	9.00	3.67	1–16
PSI***	32	84.50	86.00	17.00	53–115
Coding***	33	6.42	7.00	3.46	1–14
Symbol search***	30	7.57	7.00	3.12	1–14

*VRI* Verbal Reasoning Index, *PRI* Perceptual Reasoning Index, *WMI* Working Memory Index, *PSI* Processing Speed Index

*P < 0.05 for one sample *t* test (normative mean of 100 and SD of 15 for IQ index and normative mean of 10 and SD of 3 for subtests)

**P<0.01

***P<0.001

**Fig. 1:**
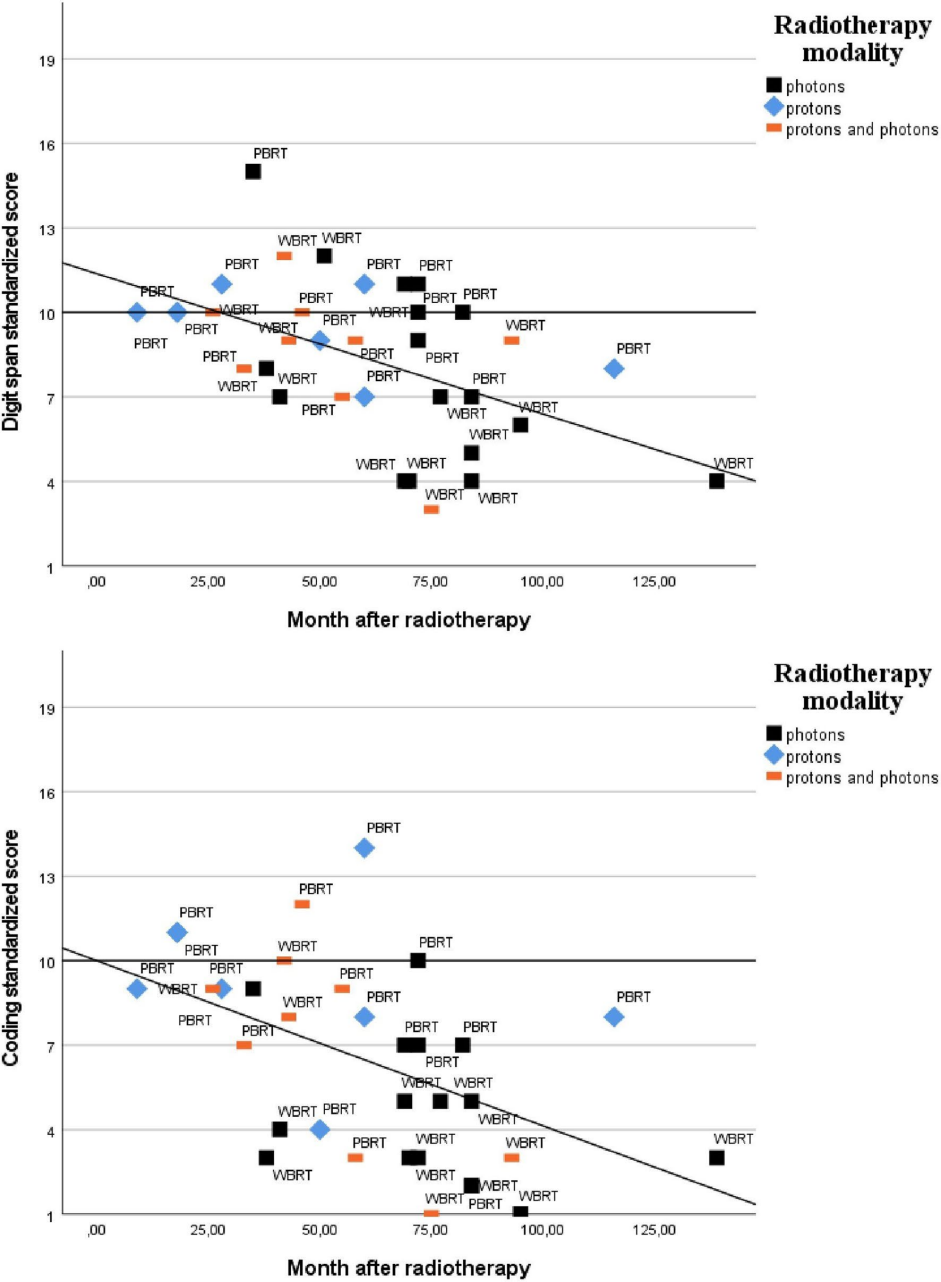
Scatterplots of performance on digit span (Working Memory) and coding (Processing Speed) (M = 10, SD = 3) against month after radiotherapy. Lower scores indicate lower performance and longer time since radiotherapy. The shapes define radiotherapy modality. WBRT is whole brain radiotherapy and PBRT is partial brain radiotherapy. Least squares regression line is shown for illustrative purposes

The time span for when the neuropsychological assessment was performed after RT varied between nine months and eleven years (139 months). Time since RT correlated moderately with lower performance on WMI (*r*_*s*_ (30) =  − 0.477, *P* = 0.008), digit span (*r*_*s*_ (34) =  − 0.509, *P* = 0.002), digit span backward (*r*_*s*_ (31) =  − 0.453, *P* = 0.011, PSI (*r*_*s*_ (32)  =  − 0.517, *p* = 0.002) and coding (*r*_*s*_ (33) =  − 0.592, *p* =  < 0.001). Correlations between time since RT and performances on digit span and coding are presented in Fig. 1.

Mean RT doses in the included OARs correlated with several IQ measurements. The correlations between mean RT dose to different brain structures and neurocognitive performance on different IQ indexes and subtests are presented in Table 3.

**Table 3:** Spearman correlation between mean radiotherapy dose to organs at risk, treatment variables and performance on IQ measurements

Cognitive measures	Cochlea right	Cochlea left	Optic nerve right	Optic nerve left	Hippocampus right	Hippocampus left	Pituitary gland	Cerebellum	Vermis	Pons	WBRT	Tumor size	Surgery
Full Scale IQ (n = 29)	− .55**	− .54**	− .48**	− .53**	− .39*	− .36	− .25	− .46**	− .50**	− .56**	− .54**	− .34	− .36*
VCI (n = 30)	− .41*	− .40*	− .39*	− .45*	− .29	− .36	− .22	− .28	− .37*	− .39*	− .39*	− .46*	− .31
Similarities (n = 33)	− .34	− .35*	− .33	− .41*	− .28	− .29	− .11	− .26	− .41*	− .37*	− .33	− .57**	− .42
Vocabulary (n = 34)	− .34*	− .34	− .37*	− .37*	− .27	− .28	− .15	− .22	− .29	− .30	− .29	− .56**	− .27
PRI (n = 30)	− .43**	− .43*	− .45**	− .43*	− .28	− .21	− .18	− .40*	− .39*	− .49**	− .44*	− .23	− .21
Matrix reasoning (n = 32)	− .46**	− .43*	− .52**	− .48**	− .37*	− .31	− .30	− .44*	− .45*	− .52**	− .43*	− .43*	− .41*
Block design (n = 32)	− .32	− .33	− .31	− .36*	− .18	− .09	− .22	− .36*	− .38*	− .42*	− .39*	− .21	− .15
WMI (n = 30)	− .53**	− .47**	− .36	− .42*	− .38*	− .33	− .20	− .52**	− .50**	− .55**	− .51**	− .32	− .52**
Digit span (n = 34)	− .38*	− .36*	− .19	− .23	− .20	− .16	− .04	− .39*	− .40*	− .36*	− .26	− .43*	− .52**
forward (n = 31)	− .20	− .14	− .10	− .12	.03	.06	.01	− .16	− .18	− .14	− .11	− .26	− .42*
backward (n = 31)	− .37*	− .40*	− .15	− .16	− .21	− .07	.04	− .43*	− .47**	− .40*	− .37*	− .38	− .43*
Letter-number-sequencing (n = 20)	− .61**	− .49*	− .46*	− .47*	− .55*	− .46*	− .33	− .44	− .55*	− .59**	− .65**	− .07	− .42*
PSI (n = 32)	− .58**	− .58**	− .53**	− .58**	− .53**	− .50**	− .36*	− .51**	− .54**	− .59**	− .62**	− .31	− .31
Coding (n = 33)	− .54**	− .54**	− .43*	− .53*	− .46**	− .47**	− .32	− .45**	− .47**	− .51**	− .51**	− .28	− .25
Symbol search (n = 30)	− .48**	− .48**	− .52**	− .53**	− .46**	− .44**	− .27	− .40*	− .43*	− .53**	− .61**	− .31	− .30

*VRI* Verbal Reasoning Index, *PRI* Perceptual Reasoning Index, *WMI* Working Memory Index, *PSI* Processing Speed Index, *WBRT *Whole Brain Radiotherapy

*P < 0.05

**P < 0.01

Bold numbers indicate significant P-values (P<0.05 and P<0.01 respectively) and moderate correlation

Mean RT doses to OARs for each child correlated with IQ measurements and the results are presented in Additional file 2: Table S2.

Mean RT doses to right and left cochleae correlated moderately with lower performance on all IQ indexes. Mean radiation doses to the right and left optic nerves correlated moderately with lower performance on FIQ, PRI, PSI and letter-number-sequencing. Mean RT dose to the left optic nerve also correlated moderately with WMI.

**Fig. 2:**
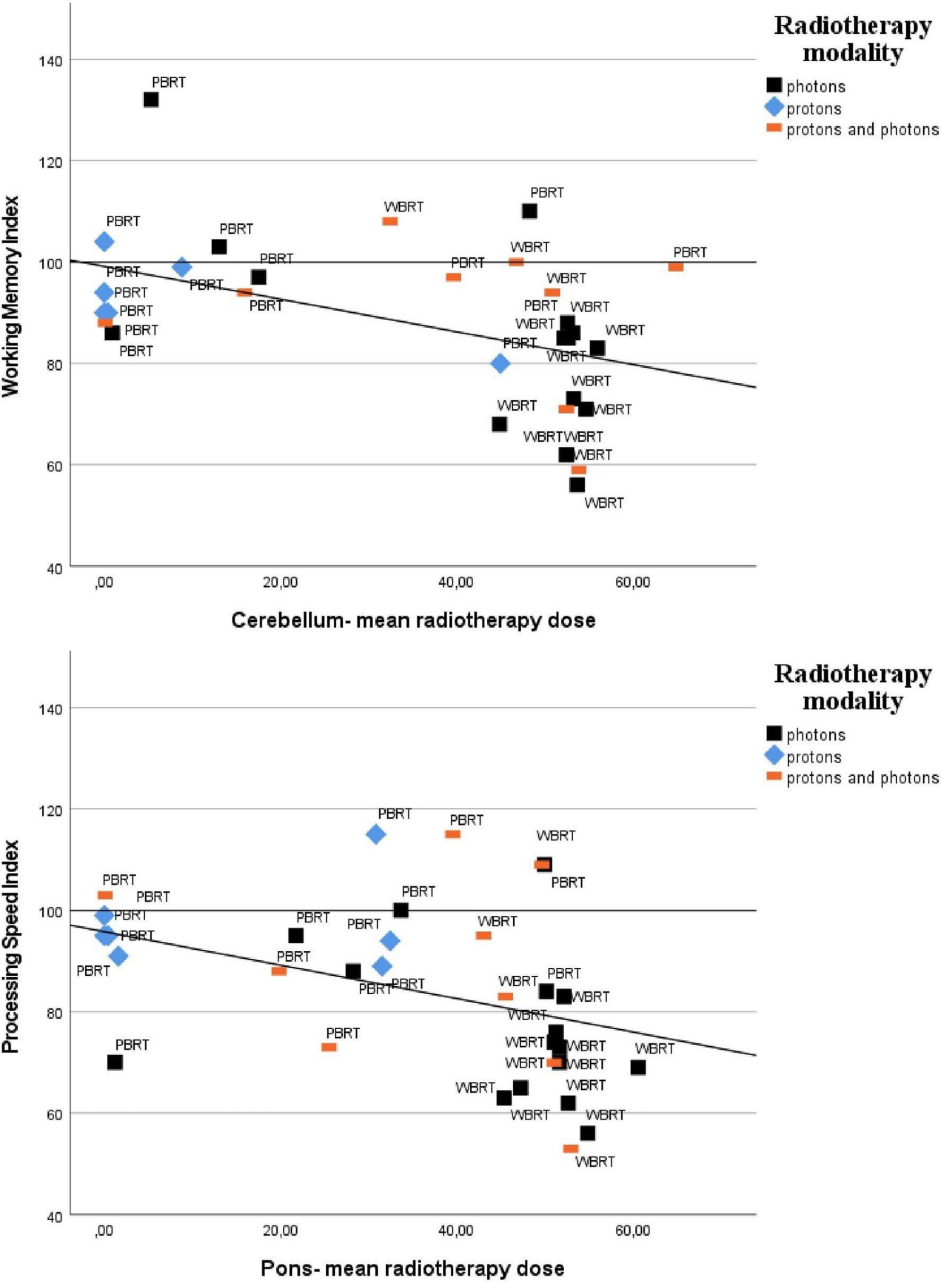
Scatterplots of performance on Working Memory Index and Processing Speed Index (M = 100, SD = 3) against mean RT dose to cerebellum and pons. The shapes define radiotherapy modality. WBRT is whole brain radiotherapy and PBRT is partial brain radiotherapy. Least squares regression line is shown for illustrative purposes

Mean RT doses in the cerebellum, vermis and pons correlated moderately with lower performance on FIQ, WMI, PSI and matrix reasoning. Mean RT doses to cerebellum and pons also correlated moderately with PRI and the vermis correlated moderately with lower performance on similarities. Figure 2 illustrates correlations between mean RT doses to cerebellum and pons with WMI and PSI.

Mean RT doses in right and left hippocampus correlated moderately with lower performance on PSI and letter-number-sequencing. Mean RT doses to chiasma, thalamus and hypothalamus (right and left) did not correlate with lower performance on IQ measurements.

When analyzing separately the group that received WBRT, mean RT dose to cerebellum and pons correlated strongly with lower performance on PRI (*r*_*s*_ (15) =  − 0.79, *P* < 0.001; *r*_*s*_ (15) =  − 0.77, *P* < 0.001), and moderately with FIQ (*r*_*s*_ (14) =  − 0.55, *P* = 0.04; *r*_*s*_ (14) =  − 0.59, *P* = 0.03). Mean RT dose to cerebellum also correlated moderately with lower performance on digit span backward (*r*_*s*_ (14) =  − 0.55, *P* = 0.04) and PSI (*r*_*s*_ (15) =  − 0.55, *P* = 0.03). Mean RT dose to pons correlated moderately with lower performance on WMI (*r*_*s*_ (14) =  − 0.57, *P* = 0.03) and mean RT to the left cochleae correlated moderately with lower performance on PRI (*r*_*s*_ (15) =  − 0.56, *P* = 0.03). Mean RT dose to the pituitary gland correlated moderately with lower performance on WMI (*r*_*s*_ (14) =  − 0.54, *P* = 0.05)*.* The lowest scores on IQ measurements were found mainly for those patients receiving whole brain RT, as expected. Total dose to the whole brain for this cohort (proton and/or photon RT only) correlated moderately with Full Scale IQ, Perceptual Reasoning Index, Working Memory Index and Processing Speed (Table 3 and Additional file 1: Table S1).

Regarding confounding treatment variables that can impact neurocognitive performance was tumor size moderately correlated with similarities, vocabulary, matrix reasoning and digit span (Table 3). When separating the result for those who received whole brain RT, tumor size did not correlate with lower performance on IQ measurements. Surgery was moderately correlated with working memory and matrix reasoning (Table 3). We found no significant correlation between chemotherapy and IQ measurements in this cohort.

## Discussion

We found a significant dose-risk relationship between mean RT dose to OARs (established and potential new OARs) and IQ subtests. Our results show that a sparing radiation dose approach, wherever possible, seems specifically critical for structures important for neurocognitive networks such as cochleae, optic nerves, hippocampus, cerebellum, vermis and pons. For those receiving whole brain RT, a sparing approach towards several brain regions that are important for neurocognitive networks would be preferable to protect the immature brain.

The cochlea is a small structure difficult to delineate, and because of this, only mean RT dose recommendations can be found in previous studies [12]. In our study, we delineated the structure with a margin to the real anatomical structure (within inner ear). Mean RT dose to cochleae is recommended to be kept below 35 Gy for children, when also synergistic toxicity of chemotherapy is considered [12]. However, there is a risk for permanent sensorineural hearing loss when cochleae are in close proximity to the target [10, 41]. Early and continuous screening for hearing impairment with appropriate management can lessen academic, language and psychosocial morbidity resulting from hearing deficit in pediatric cancer survivors [10]. IMPT as compared to IMRT can be dose-sparing to the cochleae and thus reduce the risk of IQ decline and hearing loss [11]. Our results support earlier studies pointing to the importance of reducing the RT dose to the cochleae [11, 13]. Our study extends those results by showing a significant correlation with real IQ measurements, not only with estimated IQ measurements as used previously [11]. However, further prospective studies are needed regarding reducing mean RT dose to cochlea and how this is related to sparing neurocognitive development, especially with IMPT.

In our clinical cohort no child received mean RT doses to the chiasma and optic nerve above recommended dose (46 Gy an 50 Gy) [43]*.* However, we found that a higher mean RT dose to left and right optic nerve correlated with lower performance on several IQ subtests [14, 42, 43]. The optic nerve has connections to the brain and CNS [12, 14, 15, 43] and effects on the optic nerve can be an early marker for memory loss and broad neurocognitive decline as seen in individuals with earlier stages of dementia and Alzheimer’s disease [44, 45]. Further studies are needed considering the relationship between cancer and early-onset dementia [6, 14, 46, 47], especially for brain tumor patients receiving RT and WBRT [46]. A larger mean dose reduction to the optic nerve can be achieved with IMPT, compared to IMRT [26]. To date, this is the first study relating RT dose in the optic nerves to neurocognition.

The cerebellum has an important role in neurocognition [19], and our results are in line with a previous study suggesting that the cerebellum and vermis may be defined as OARs [19, 20]. The pons has important connections with the cerebellum [48] and we found similar correlations for IQ indexes and mean RT dose to the pons, as for mean RT dose to the cerebellum. Mean RT dose was the highest to the pons and recent studies have stressed the importance of lowering the recommended dose to the brainstem, especially for proton RT [49, 50]. However, with strict brainstem dose constraints a low risk of injury has been found with proton RT [51]. In line with this, our study suggests that more conservative dose restrictions should be considered when RT is given to the pons.

Our results also highlight the importance of limiting and/or avoiding the RT dose in the hippocampus to preserve working memory and processing speed [12, 23, 24]*.* A substantial reduction in mean dose in the hippocampus can be achieved with proton RT, compared to photon RT [52]. With hippocampal sparing IMPT it can be possible to reduce the mean dose to the hippocampus considerably (about 20 Gy_RBE_) with minimal impact on whole-brain target and with an estimated reduced risk of neurocognitive impairment [53]. Although, to a lesser extent, with VMAT a mean RT dose reduction of 50% of the prescribed dose to the planning target volume can be achieved with photon RT during WBRT [29].

A sparing approach to the hypothalamus/pituitary gland is important to avoid HP disorders [26, 29] that have been associated with neurocognitive decline [22]. We found a significant dose-risk relationship between the pituitary gland and working memory that supports the importance of a sparing approach. A dose reduction can be achieved with both IMPT [26] and VMAT [29]. In our clinical cohort many children received doses above 25–30 Gy that are now the upper recommended dose limits [12]. However, more studies are needed to further investigate the RT-induced neurocognitive effect on the hypothalamus, thalamus and pituitary gland that also include other neurocognitive measures than IQ, such as visual attention and fatigue. IQ measurement is a common and good psychometric measure for RT-induced neurocognitive decline, especially for those receiving WBRT [28]. However, IQ measurements alone are not sufficient to detect all RT-induced neurocognitive decline and more comprehensive neuropsychological assessment is needed [7] and will be investigated in our upcoming long-term study.

There are several other confounding factors that can influence neurocognitive performance that we have presented in our previous study [7]. In this study we excluded those who received gamma knife only and presented results on some potential confounding treatment variables and association with neurocognition. Tumor size and surgery correlated with some IQ measurements and interactions effects needs to be considered. Still, mean RT dose towards OARs was highly significant correlated with IQ measurements and further explain neurocognitive decline. In the present study, we further evaluated RT-induced neurocognitive decline with a more precise measure and physical mean dose metrics is especially suitable for heterogeneous samples. In our previous study we found that planning target volume was correlated with letter-number-sequencing only [7]. These findings strengthen the notion that physical mean dose metrics seem to be a more precise predictor for RT-induced neurocognitive decline [8].

However, there are several limitations in this study due to the heterogeneity with different diagnoses, treatments and time points of neuropsychological follow-up. We could not explore interaction effects on different RT modalities and different risk factors from a multivariable approach. Delineation of OAR is a critical step to treatment planning and OARs were outlined according to available atlas guidelines [30, 31]. However, there is a heterogeneity in normal structures contouring between professionals and to be able to replicate these findings and to further decrease inter-and intra- observer OAR delineation variability, updated neuro-contouring atlases is essential [42, 54, 55], as would more precise measures be, ideally using newer software that incorporates linear energy transfer (LET) or variable RBE weighted doses [56].

Even though, this study highlights the importance of further studies to corroborate these results. Replication of these findings in a larger more homogenous sample is needed with updated contouring and analysis of doses to different OARs. Comprehensive neuropsychological assessments are also needed to detect core neurocognitive functions [7]. Systematic and standardized neuropsychological follow-up before and after RT at specific time points is highly recommended [7, 57–59]. This is also important for other diagnoses receiving high RT doses to different brain structures important for neurocognitive networks, such as head and neck cancer [60]. Potential confounding risk factors also need to be identified early and followed over time since several factors can interact, such as other treatments variables, the tumor itself and neurological symptoms, as reported in previous study [7]. The interplay between various risk factors such as physical and psychosocial factors, school attendance and rehabilitation interventions can also enhance or limit neurocognitive development [61].

For systematic evaluation of clinical outcomes and to address for larger cohorts, medical, neurocognitive and educational data needs to be systematically collected in national and/or international quality registries [56, 62, 63]. For proton RT there is a need for collaboration between clinical proton centers to establish common platforms and perspectives for optimization and treatment planning evaluation and actively help developing methods and tools for clinical implementation of the more complex metrics [64]. Since august 2015 all children in Sweden are offered proton RT at the Skandion clinic in Uppsala, without additional costs for the families [65], if this treatment is preferable. Even though proton RT often is preferable, it is not available or equally accessed for all cancer patients who need it throughout the world [57]. The relationship between doses to critical brain structures important for neurocognitive networks still needs to be evaluated regardless of RT modality. To explore possibilities to limit mean RT doses toward OARs, and thus to reduce neurocognitive sequelae, larger long-term follow-up multicenter studies and homogenous study designs with different research questions are essential.

## Conclusion

Highly significant correlations were found between mean RT dose to vulnerable brain structures and neurocognition. Taken together, the results show that a sparing radiation dose approach, wherever possible, seems specifically critical for structures important for neurocognitive networks, such as cochleae, optic nerves, hippocampus, cerebellum, vermis and pons.

### Supplementary Information

**Additional file 1: Table S1.** Clinical characteristics of 44 eligible patients.

**Additional file 2: Table S2.** Mean radiotherapy doses to organs at risk.

## Data Availability

The dataset that supports the findings of this study is available from the corresponding author, [H.S], upon reasonable request.

## Abbreviations

RT: Radiotherapy
OAR: Organ at risk
FIQ: Full-scale IQ
PRI: Perceptual Reasoning Index
WMI: Working Memory Index
PSI: Processing Speed Index
WBRT: Whole Brain Radiotherapy
CNS: Central nervous system
HP: Hypothalamic-pituitary
IMPT: Intensity-modulated proton therapy
IMRT: Intensity-modulated radiation therapy
VMAT: Volumetric-modulated arc therapy
CT: Computed tomography
Gy: Gray
RBE: Relative biological effectiveness
VRI: Verbal Reasoning Index
SD: Standard deviation
SS: Standardized scores
*r*_*s*_: Spearman’s rank correlation coefficient
PBRT: Partial brain radiotherapy
