# Supplementary material for: Neurocognition and mean radiotherapy dose to vulnerable brain structures: new organs at risk?
Source: Radiat Oncol. 2023 Aug 11;18:132. doi: 10.1186/s13014-023-02324-2 (PMC10416465; doi:10.1186/s13014-023-02324-2)
Supplement: Supplementary file 1 — Additional file 1: Table S1. Clinical characteristics of 44 eligible patients. [file 13014_2023_2324_MOESM1_ESM.docx]

**Additional file 1: Table S1.** Clinical characteristics of 44 eligible patients.

| P | Diagnosis WHO | Sex | Age  RT | Tumor location | IICP | S | Chemotherapy | RT | WBRT  (Gy) | PTV  (GY) |
| --- | --- | --- | --- | --- | --- | --- | --- | --- | --- | --- |
| 1  2  3  4  5  6  7  8  9  10  11  12  13  14  15  16  17  18  19  20  21  22  23  24  25  26  27  28  29  30  31  32  33  34  35  36  37  38  39  40  41  42  43  44 | Medulloblastoma  Medulloblastoma  Medulloblastoma  Medulloblastoma  Medulloblastoma  Medulloblastoma  Medulloblastoma  Medulloblastoma  Medulloblastoma  Medulloblastoma  Medulloblastoma  Medulloblastoma  Medulloblastoma  Medulloblastoma  ETMR*  Pineoblastoma  Pilocytic astrocytoma, grade 1-2  Pilocytic astrocytoma, grade 3-4  Pilocytic astrocytoma, grade 1  Pilocytic astrocytoma, grade 1  Pilocytic astrocytoma, grade 3  Pilocytic astrocytoma, grade 1  Pilocytic astrocytoma, grade 1  Papillary ependymoma, grade 2  Papillary ependymoma, grade 2  Ependymoma, grade 3  Anaplastic ependymoma, grade 3  Ependymoma, grade 2-3  Ependymoma, grade 2  Anaplastic ependymoma, grade 3  Anaplastic ependymoma, grade 3  Anaplastic ependymoma, grade 3  Craniopfaryngioma  Craniopfaryngioma  Craniopfaryngioma  Germinoma  Germinoma  Germinoma  Germinoma  Germinoma  Germinoma  ONSM**, Schwannoma, Nf2***  ONSM  Unclassified | F  M  M  F  F  M  F  M  F  F  M  M  M  F  M  F  M  F  M  F  F  M  M  F  M  F  M  M  M  M  F  F  M  F  M  M  F  M  M  M  F  M  F  F | 9  11  6  10  8  9  9  6  11  10  6  11  7  6  5  14  5  17  10  16  11  8  6  14  5  12  7  9  16  3  17  8  14  7  17  15  15  13  13  17  7  13  15  9 | Cerebellum (vermis)  Cerebellum  Cerebellum  Cerebellum (fourth ventricle)  Cerebellum (vermis)  Cerebellum (vermis)  Cerebellum (vermis)  Cerebellum  Cerebellum (third ventricle)  Cerebellum  Cerebellum (fourth ventricle)  Cerebellum (fourth ventricle)  Cerebellum (fourth ventricle)  Cerebellum (vermis, fourth ventricle)  Cerebrum (parieto-occipital left)  Corpus pineale  Cerebellum right (vermis)  Cerebrum (fronto-parietal right)  Hypothalamus  Hypothalamus  Cerebellum  Pituitary gland and hypothalamus  Cerebellum  Cerebrum (fronto-parietal left)  Cerebrum (fronto-parietal left)  Cerebellum (fourth ventricle)  Cerebrum (parieto-occipital left)  Cerebellum (left)  Foramen Monro  Cerebellum (vermis)  Cerebrum (frontal left)  Cerebrum (frontal right)  Pituitary gland and Sella Turcica  Pituitary gland  Pituitary gland and hypothalamus  Corpus pineale  Hypothalamus  Corpus pineale  Corpus pineale  Corpus pineale  Hypothalamus, chiasma, optic tracts  Multifocal  Optic tract (right)  Corpus pineale | Yes  Some  Yes  Yes  Yes  Yes  Yes  Yes  Yes  Yes  Some  Yes  No  Yes  Yes  Yes  No  No  Some  Yes  No  Yes  No  No  No  Yes  Yes  No  Some  Yes  No  No  No  No  No  Yes  No  Some  Yes  Yes  No  Yes  No  Yes | T  T  T  T  T  T  T  P  M  T  T  P  T  T  P  B  B  M  P  B  T  P  P  P  T  M  P  P  T  M  T  T  M  P  P  B  N  B  B  P  N  N  N  N | Eto, Car, Vin, Cis, Cyc, CCNU  Eto, Car, Vin, Cis, Cyc, CCNU  Eto, Car, Vin, Cis, Cyc, CCNU  Vin, Cis, CCNU  Vin, Cis, CCNU  Vin, Cis, CCNU  Car, Eto, Ifo  Vin, Cyc, Cis, CCNU  Eto, Car, Vin, Cis, Cyc, CCNU  Vin, Cyc, Cis, CCNU  Vin, Cyc, Car, Eto  Vin, Cis, CCNU  Vin, Cis, CCNU  Vin, Cyc, Met, Car, Tem  Eto, Car, Vin, Cis, Cyc, CCNU  Eto, Car, Vin, Cis, Cyc, CCNU  Vin, Car  Tem  Vin, Car, Vinb  Vin, Car  Tem  Vin, Car  Vin, Car  No  Vin, Cis, CCNU  Vin, Cyc, Car, Eto  No  Vin, Cyc, Car, Eto  No  Vin, Car, Cyc, Cis  No  Vin  No  No  No  Car, Eto, Ifo  Car, Eto, Ifo  Car, Eto, Ifo  Car, Eto, Ifo  Cis, Eto, Ifo, Car  Car, Eto, Ifo  No  Vin, Cyc, Met, Car, Eto  Car, Eto | Ph  Ph  Ph  Ph  Ph  Ph  P and Ph  Ph  Ph  Ph  P and Ph  Ph  P and Ph  P and Ph  Ph  P and Ph  Ph  Ph  P  P  P and Ph  P  P and Ph  Ph  Ph  Ph  Ph  Ph  P  P  P  P and Ph  P  P  P  Ph  P and Ph  Ph  P and Ph  P and Ph  Ph  P and Ph  P  P | 35  35  35  23  23  23  23  35  23  23  23  40  35  23  35  36  24 | 55  55  55  54  56  54  54  54  54  54  54  60  55  56  55  59  54  60  50  54  56  51  54  56  54  54  55  54  54  54  53  57  54  54  54  42  40  40  40  40  24  50  50  54 |

Abbreviations: P, Patients; F, Female; M, Male; IICP, Increased Intracranial Pressure; S; Surgery; T, Total resection; P, Partial resection; M: Multiple resection; Eto, Etoposide; Car, Carboplatin; Vin, Vincristine; Cis, Cisplatin; Cyc, Cyclophosphamide; CCNU, Lomustine; Ifo, Ifosfamide; Tem, Temozolamide; Vinb, Vinblastine; Met, Methotrexate; RT, Radiotherapy; Ph, Photon; P, Proton; G, Gamma; WBRT, Whole Brain Radiotherapy, PTV; Planning target volume

* Embryonal tumor with multilayer rosette

** Optic Nerve Sheath Meningioma

*** Neurofibromatosis type 2
