# Supplementary material for: Neurocognition and mean radiotherapy dose to vulnerable brain structures: new organs at risk?
Source: Radiat Oncol. 2023 Aug 11;18:132. doi: 10.1186/s13014-023-02324-2 (PMC10416465; doi:10.1186/s13014-023-02324-2)
Supplement: Supplementary file 2 — Additional file 2: Table S2. Mean radiotherapy doses to organs at risk. [file 13014_2023_2324_MOESM2_ESM.docx]

**Additional file 2: Table S2.** Mean radiotherapy doses to organs at risk.

| P | RT | Cochlea  right | Cochlea  left | Optic nerve  right | Optic nerve  left | Hippo-  campus  right | Hippo-  campus left | Pituitary  gland | Cerebellum | Vermis | Pons |
| --- | --- | --- | --- | --- | --- | --- | --- | --- | --- | --- | --- |
| 1  2  3  4  5  6  7  8  9  10  11  12  13  14  15  16  17  18  19  20  21  22  23  24  25  26  27  28  29  30  31  32  33  34  35  36  37  38  39  40  41  42  43  44 | Ph  Ph  Ph  Ph  Ph  Ph  P and Ph  Ph  Ph  Ph  P and Ph  Ph  P and Ph  P and Ph  Ph  P and Ph  Ph  Ph  P  P  P and Ph  P  P and Ph  Ph  Ph  Ph  Ph  Ph  P  P  P  P and Ph  P  P  P  Ph  P and Ph  Ph  P and Ph  P and Ph  Ph  P and Ph  P  P | 51,60  55,40  49,80  52,00  53,90  51,30  24,10  51,90  47,90  35,60  25,40  46,10  50,50  23,30  39,20  29,30  16,40  1,10  ,20  17,10  38,20  3,20  3,50  1,00  ,90  21,00  3,20  20,40  ,00  3,80  ,30  ,20  ,00  13,50  ,05  10,90  1,50  8,30  4,30  44,00  12,70  5,00  ,00  2,80 | 51,10  55,20  52,30  52,20  55,70  54,10  23,30  52,20  35,90  35,60  25,09  45,50  48,90  26,30  48,30  28,90  14,90  1,20  ,20  14,90  15,00  3,20  5,20  1,10  ,80  22,80  2,40  49,80  1,10  1,00  ,40  ,10  ,00  1,10  7,40  10,90  8,70  4,60  4,30  43,70  8,90  10,20  ,00  2,40 | 27,10  31,70  26,30  19,60  36,30  25,00  17,40  39,20  34,80  23,80  22,80  36,10  29,20  18,90  21,20  29,20  3,20  ,80  33,90  10,90  3,70  17,10  ,10  1,10  1,10  8,90  5,00  7,20  ,00  ,10  ,40  ,70  12,40  31,30  1,20  1,40  4,80  6,70  2,10  33,00  ,00  1,20  8,80  4,30 | 29,80  28,40  30,60  19,50  36,00  29,50  16,80  37,40  24,50  23,80  23,50  34,90  31,70  20,10  32,40  28,40  3,10  ,80  15,40  6,90  2,70  22,70  ,10  1,30  ,90  8,20  3,30  7,40  ,00  ,10  ,50  ,30  9,70  27,50  ,30  1,40  3,70  11,60  2,90  31,90  13,00  3,40  ,60  3,20 | 51,20  54,40  40,10  50,50  38,50  39,10  32,60  48,10  39,90  37,70  32,63  45,80  51,60  26,50  41,10  44,60  52,40  7,60  22,80  20,10  17,50  26,70  6,90  8,10  2,00  34,10  40,40  21,90  1,60  11,90  1,00  1,00  ,30  21,60  15,20  24,60  28,70  31,40  31,60  50,90  22,00  ,40  ,00  33,70 | 51,50  54,60  43,30  50,50  41,40  42,90  35,30  51,10  43,30  39,30  32,50  45,10  48,20  28,80  51,30  45,40  52,40  5,00  9,47  20,30  9,90  23,60  5,80  1,90  1,50  36,10  20,10  42,30  ,30  12,20  1,50  ,10  ,00  43,40  24,80  25,50  25,60  36,10  27,70  51,60  22,70  ,40  ,00  34,70 | 33,40  35,10  32,80  36,90  37,60  46,60  22,60  50,70  36,60  26,30  22,50  42,40  48,20  22,40  41,20  29,40  42,00  1,40  48,70  51,50  5,00  48,20  ,20  1,50  1,40  25,00  4,50  15,20  ,05  ,30  ,60  ,20  52,10  53,40  49,10  13,70  40,60  25,20  15,90  43,80  22,80  6,30  ,20  44,40 | 53,20  55,90  53,10  52,40  54,60  53,60  50,80  51,90  52,60  52,10  46,70  54,50  52,40  53,80  44,80  32,40  17,50  2,00  ,80  ,20  64,80  2,90  39,60  1,20  ,90  52,50  10,70  48,20  ,00  44,90  ,40  ,10  ,00  ,00  9,09  16,40  15,70  5,30  15,90  41,70  13,00  8,80  ,00  8,80 | 52,60  55,60  52,80  52,70  53,90  53,30  52,70  51,90  51,90  51,70  53,20  53,30  52,30  54,50  47,00  34,60  26,00  3,40  1,40  ,20  52,60  2,10  45,80  1,80  1,20  52,20  22,20  51,70  ,00  50,10  ,60  ,10  ,00  ,00  14,10  17,70  16,20  27,60  19,20  45,30  17,90  7,70  ,00  14,10 | 60,50  54,80  47,20  51,60  52,10  52,60  49,60  51,60  51,20  51,00  45,50  52,20  51,00  52,90  45,30  43,00  28,20  1,70  10,10  15,10  39,50  31,50  25,50  1,44  1,20  50,10  7,30  49,90  ,00  32,40  ,42  ,10  1,60  14,30  30,00  20,70  23,40  33,60  19,80  48,00  21,70  13,00  ,00  30,80 |

Abbreviations: P, Patients; RT, Radiotherapy^[[1]](#footnote-1)^

1. All children receiving RT 2003-2007 were delineated and dose-calculated in Treatment Management system (TMS). For children receiving RT 2008-2015, 15 were delineated and dose-calculated in TMS, 15 in Oncentra and five in both TMS and Oncentra. All children receiving proton RT were delineated and dose-calculated in TMS. [↑](#footnote-ref-1)
